# Intelligent Design Optimization System for Additively Manufactured Flow Channels Based on Fluid–Structure Interaction

**DOI:** 10.3390/mi13010100

**Published:** 2022-01-08

**Authors:** Haonan Ji, Bin Zou, Yongsheng Ma, Carlos F. Lange, Jikai Liu, Lei Li

**Affiliations:** 1 Center for Advanced Jet Engineering Technologies (CaJET), School of Mechanical Engineering, Shandong University, Jinan 250061, China; 201913907@mail.sdu.edu.cn (H.J.); zb78@sdu.edu.cn (B.Z.); jikai_liu@sdu.edu.cn (J.L.); 2 Key Laboratory of High-Efficiency and Clean Mechanical Manufacture at Shandong University, Ministry of Education, Jinan 250061, China; 3 National Demonstration Center for Experimental Mechanical Engineering Education, Shandong University, Jinan 250061, China; 4 Department of Mechanical and Energy Engineering, Southern University of Science and Technology, Shenzhen 518055, China; mays@sustech.edu.cn; 5 Department of Mechanical Engineering, University of Alberta, Edmonton, AB T6G 1H9, Canada; carlos.lange@ualberta.ca

**Keywords:** expert system, fluid–structure interaction, design optimization, additive manufacturing, flow channel

## Abstract

Based on expert system theory and fluid–structure interaction (FSI), this paper suggests an intelligent design optimization system to derive the optimal shape of both the fluid and solid domain of flow channels. A parametric modeling scheme of flow channels is developed by design for additive manufacturing (DfAM). By changing design parameters, a series of flow channel models can be obtained. According to the design characteristics, the system can intelligently allocate suitable computational models to compute the flow field of a specific model. The pressure-based normal stress is abstracted from the results and transmitted to the solid region by the fluid–structure (FS) interface to analyze the strength of the structure. The design space is obtained by investigating the simulation results with the metamodeling method, which is further applied for pursuing design objectives under constraints. Finally, the improved design is derived by gradient-based optimization. This system can improve the accuracy of the FSI simulation and the efficiency of the optimization process. The design optimization of a flow channel in a simplified hydraulic manifold is applied as the case study to validate the feasibility of the proposed system.

## 1. Introduction

**Figure 1:**
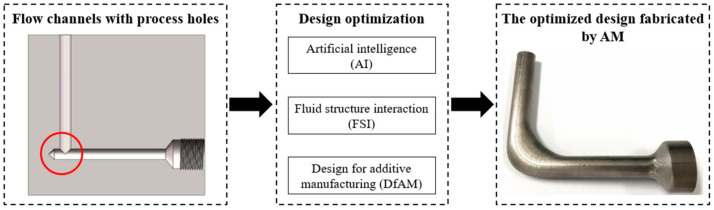
The traditional right-angle flow channel optimized by the intelligent design optimization system and fabricated by AM.

Flow channels are the core structures in hydraulic components. The traditional flow channels are usually right-angle channels with process holes due to the limitations of processing and design methods, which causes the appearance of vortices and energy loss of the flow [1]. To solve this problem effectively, it is essential to seek a kind of superior processing technology and optimize the flow channels [2] inside the hydraulic components accordingly [3]. On one hand, additive manufacturing (AM) has been increasingly applied in the processing of the flow channels because it can manufacture parts of any shape. In order to fully take advantage of AM, it is necessary to incorporate design for additive manufacturing (DfAM) methodology [4] into the optimization of flow channels. On the other hand, the simulation of flow channels is essential to gain an insight into the design optimization, in which Computational Fluid Dynamics (CFD) is commonly applied in the analysis of the flow field [5]. When the pressure of the fluid domain causes the deformation of the solid domain, the fluid domain will be affected correspondingly. In this case, the fluid–structure interaction (FSI) should be considered, otherwise the accuracy of the calculation results cannot be guaranteed. Multiple sets of FSI simulation results can provide the data foundation for the establishment of the mathematical model, and the design optimization of flow channels can be derived by optimization algorithms. However, hydraulic components with flow channels are usually featured with complex modeling schemes, which require costly effort from highly experienced modelers. As a result, the acquisition of simulation data is a tedious and error-prone process. Therefore, an optimization system for AM flow channels based on artificial intelligence (AI) is demanded to setup simulation, integrate the heterogeneous modules, and process data. With the assistance of such a system, the optimization of flow channels can be realized, and the optimized design can be fabricated by AM, as shown in Figure 1.

In this paper, an intelligent design optimization system based on expert system [6] theory is proposed. This system is a general system with the flow channel structure as the optimization object. To reduce the computation load, the multi-channel structure can be disassembled into several single channels by focusing on the optimization of typical features, such as right-angle channels. In this system, the modeling scheme and design parameters should be firstly defined based on the characteristics of directed energy deposition (DED) and optimization goals. According to the model features [7] and initial conditions, the intelligent allocation of fluid physics models is achieved, and the accurate CFD simulation results of different design points are obtained after computation. Based on the CFD results, the design optimization of flow channels under constraints is conducted to achieve the optimization objectives. The pressure distribution of the optimized flow field is coupled to the corresponding solid interface, so that the structural strength analysis is performed. Subsequently, the intelligent optimization algorithm is applied to derive the optimal value of the objective function under constraints with design parameters and the corresponding FSI results as the data input. Specifically, the research on CFD- and FSI-based optimization is reviewed in Section 2. The principles and implementation of intelligence in FSI applied in this paper are presented in Section 3. The architecture and function of the intelligent design optimization system are introduced in Section 4. Following that, a case study of a flow channel in a hydraulic manifold is demonstrated in Section 5 to verify the effectiveness of the proposed system.

## 2. Literature Review

Design optimization is commonly applied in the improvement of complex parts involving fluid flow. In order to ensure accurate data support, FSI is the preferred simulation method in such kind of scenarios. However, design optimization usually requires a large amount of simulation data, so it is necessary to adopt intelligent approaches to improve the efficiency and accuracy of simulation. After design optimization, AM is suitable for the fabrication of the optimized flow channels due to its capability in producing complex structures. The following sub-sections offer a detailed review of the relevant aspects.

### 2.1. Intelligence in FSI Simulation

In many physical models which have solid and fluid domains, there is an interaction between each domain due to the displacement and deformation of the solid structures under the flow influence [8]. For this kind of multi-domain interaction problem, called multiphysics applications, it is almost impossible to solve only with the knowledge of a single discipline [9]. Therefore, it is necessary to consider the influence between the fluid domain and the solid domain through FSI. For different interaction situations, FSI is usually divided into one-way coupling and two-way coupling [10,11,12]. The difference between these two methods of coupling lies in if the information in the fluid domain and the solid domain are transmitted to each other. Among them, the two-way coupling of FSI includes the strong two-way coupling and the weak two-way coupling. The strong two-way coupling problems require solving equations of the fluid domain and solid domain simultaneously, while the equations of the fluid domain or solid domain should be solved firstly and then the data is exchanged in subsequent calculations for the weak two-way coupling [13]. No matter which method is adopted, the foundation is the conservation of physical variables such as stress and temperature on the interface between fluid–structure (FS) domains.

The simulation objects of FSI include the solid domain and fluid domain, and the solution of the fluid domain depends on CFD [12]. For components involving internal fluid flow, a large number of CFD simulations are necessary for the design optimization of flow channels due to their complex structures [14], which requires significant experience and time input. To alleviate this issue, CFD simulations are in urgent need for intelligence. One approach is to apply AI in CFD solvers to improve the solving efficiency. Boosari et al. [15] designed a fast-track data-driven method based on artificial neural networks (ANNs). The computational speed of the model was increased by 100 times compared to traditional CFD by using the proposed intelligent computing model, which was proved by the two-dimensional dam-break. The other approach is to apply AI to predict the flow field. Studies have shown that prediction models can be generated by intelligent measures to predict a variety of fluid phenomena that may occur in flow channels, thus effectively replacing CFD and greatly saving the time of numerical simulation [16]. Shamshirband et al. [17] forecasted the erosion rate of micro- and nano-sized particles in a 90° elbow based on an adaptive neuro-fuzzy inference system (ANFIS). Compared with CFD, the ANFIS prediction model had higher calculation accuracy under the verification by root-mean-square error (RMSE). Lee et al. [18] proposed a CFD-based multi-resolution simulation framework to resolve multiscale problems. The approximation theory and domain decomposition were combined with statistical techniques, such as the co-kriging method, to predict the result. Ren and Cao [19] proposed a low-dimensional data transformation model through the CFD-based open-source platform OpenFOAM, which can improve the efficiency of the CFD-based prediction model while ensuring simulation accuracy.

At present, research on the intelligence of FSI simulation is mainly focused on building prediction models to replace the CFD solver by learning substantial data with a variety of intelligent algorithms. However, the accuracy of the prediction model is usually not as high as the original solver, leading to larger errors when constructing approximate fitting equations. The flow regime in the complex channel is mostly turbulent, which makes it difficult to configure the simulation, and it has a high dependence on expert knowledge. Therefore, the key problem in FSI simulation is how to complete the configuration of CFD accurately and intelligently without human intervention, thereby providing data support for design optimization more efficiently.

### 2.2. FSI-Based Design Optimization

With the wide application of FSI in the simulation of hydraulic components, the combination of FSI technology and multiple design optimization methods has been studied. FSI is one of the main methods to obtain the data needed for parametric optimization. For example, Kang and Kim [20] conducted research on response surface methodology (RSM)-based and FSI-based optimal design methods for a centrifugal compressor impeller by using ANSYS DesignXplorer (DX). It was demonstrated that the structural safety of the impeller could be improved by 10% at the maximum stress. Wen et al. [21] optimized the fin structure comprehensively through the multi-objective Genetic Algorithm (MOGA) based on FSI and RSM. A multi-objective optimization that increased the heat transfer efficiency and decreased the maximum stress was established. As a robust and capable tool [22], topology optimization can be combined with FSI to improve the design. Combining the level set method (LSM) and the extended finite element method (XFEM), Jenkins and Maute [23] presented an FSI-based topology optimization approach. To solve the optimization problems based on the steady-state FSI, Yoon [24] developed a new stress-based topology optimization method (STOM) based on the qp-relaxation method and the global p-norm approach, which can acquire the structure with the minimum volume under the local stress constraint. Furthermore, the shape optimization can be combined in the process of fluid–structure coupling solution. Jang et al. [25] proposed a design optimization method to solve highly nonlinear multi-physics problems, which can be applied to the reliable optimization of the solid domain affected by FSI. The feasibility of the proposed method is verified by the shape optimizations of a channel and a vessel. Aghajari and Schafer [26] presented an FSI-based quadratic sequential programming algorithm to accelerate the process of shape optimization. In addition, adjoint shape optimization is an important approach involving FSI, which reduces the computation of multivariable optimization by calculating adjoint equations [27,28]. However, the sensitivity information is difficult to obtain in complex flow scenarios, which in turn results in a high computational cost. [29].

Although some research cases of FSI applied in various optimization methods have been studied, and several of them also applied the intelligent algorithm to improve the performance of calculation, there still lacks research of systematic FSI-based optimization. It is necessary to build an intelligent design optimization system to integrate a series of cumbersome functions, including running each group of the FSI simulation intelligently, recording the information automatically, and calculating the optimal value by optimization algorithms. The characteristics and constraints of AM should also be considered in the design optimization to meet the actual manufacturing requirements.

### 2.3. AM in the Fabrication of Optimized Flow Channels

The optimized flow channels usually eliminate the process holes due to the drilling operation and replace them by other transition forms at the intersection of two or more flow channels. Faced with such kinds of complex structures, the traditional machining method has the disadvantages of intractable processing, low efficiency, and waste of materials. In comparison, AM can make intricate parts of any shape by adding materials layer by layer [30,31]. When parts have complex features such as curved surfaces and bending angles, the advantages of AM can be adequately reflected, making it a suitable manufacture solution to the optimized flow channels. Zhang et al. [32] replaced the process holes by the smooth transition in flow channels of a hydraulic manifold and manufactured the optimized hydraulic manifold by a stereo lithography apparatus (SLA). Through selective laser melting (SLM), Alshare et al. [33] produced a hydraulic manifold that was redesigned based on FSI simulations. Many studies on the lightweight design of flow channels only utilized the characteristics of AM; however, it is necessary to add the process constraints of AM into the design optimization of flow channels considering their complex situations in actual machining. To improve the comprehensiveness of design optimization, the concept of DfAM needs to be introduced. DfAM is proposed to take full advantage of the manufacturing capacity to achieve better performance while meeting the requirements of AM, which requires the designers to add AM direction, support structures, size limitations, and so on, as the design constraints [34,35,36]. For instance, Biedermann et al. [37] proposed a CAD-based design method for AM flow channels to meet the AM overhang constraint. This method can avoid most support structures by changing the cross-section shape and the AM direction. Ponche et al. [38] presented a new numerical DfAM methodology that can optimize the geometries of products based on the product performance and the process requirements. The feasibility of the method was verified by a turbine blade. In addition, different AM technologies are featured with various processing factors such as forming principles, direction, materials, and so on; hence, the design constraints should be set in DfAM for each AM technology, respectively [34].

Based on the literature reviewed, DfAM of flow channels is mainly focused on how to exert the advantages of AM and reduce the support structure in the manufacturing. Unlike the aforementioned AM techniques, the cradle five-axis DED technology adds an A-axis and C-axis on the basis of a three-axis machine tool, which can keep the overhang angle within a reasonable range. The rotation of the platform around the X-axis and Z-axis offers the freedom to achieve complex forming methods, such as changing AM direction, printing on curved surfaces, and so on; thus, flow channels can be printed along its axis, and support structures can be avoided. However, the design of flow channels manufactured by this technique has been rarely studied. Therefore, it is of practical significance to integrate various processing constraints of DED, such as the rotation angle of the turntable, the minimum wall thickness, and so on, into the DfAM of flow channels, which can maximize the AM capability in manufacturing flow channels with improved performance.

## 3. Intelligent FSI Simulation System

FSI, widely applied to various engineering research projects, is a technical means of considering the interaction between FS domains. Instead of solving the domains independently, the essence of FSI is solving sets of equations that are established by the mathematical expressions of hydromechanics and solid mechanics. The arbitrary Lagrangian Eulerian (ALE) descriptive equations for the incompressible Newtonian fluid flow are as follows:

(1) $$\rho_{f}\frac{\delta v_{f}}{\delta t}+\rho_{f}\left(v_{f}-{\hat{v}}_{f}\right)\cdot \nabla v_{f}=\nabla \sigma_{f}+f^{B},\;\mathrm{in}\;\Omega_{f}$$

(2) $$\nabla \cdot v_{f}=0,\;\mathrm{in}\;\Omega_{f}$$

(3) $$\sigma_{f}=-p_{f}I+\mu [\nabla v_{f}+{\left(\nabla v_{f}\right)}^{T}],\;\mathrm{in}\;\Omega_{f}$$

where the domains are described as Ω_f_ and Ω_S_, *ρ*_f_ and *v*_f_ are the density and velocity of the fluid, respectively, *t* is time, the symbol ▽ is used to denote the divergence and gradient operators, ${\hat{v}}_{f}$ is the velocity of the moving ALE frame, *σ*_f_ is the fluid stress tensor, *f*^B^ is the vector of fluid forces, *I* is the identity tensor, *μ* is the fluid viscosity, and *p*_f_ is the fluid pressure. The Lagrangian equations for the solid structure are

(4) $$\rho_{s}\frac{\partial v_{s}}{\partial t}=\nabla \cdot \sigma_{s}+f^{B},\;\mathrm{in}\;\Omega_{s}$$

(5) $$\det(F)=1,\;\mathrm{in}\;\Omega_{s}$$

(6) $$\sigma_{s}=G(F\cdot F^{T}-I)-p_{s}I,\;\mathrm{in}\;\Omega_{s}$$

where *ρ*_s_ and *v*_s_ are the density and velocity vector of the solid displacement respectively, *F* is the deformation tensor, *G* is the solid shear modulus, *σ*_s_ is the Cauchy stress tensor, and *p*_s_ is the fluid pressure [10].

The flow channel structure in this paper has strength constraints during the design optimization process. The deformation of the solid domain of the flow channel is generally so small that the influence on the fluid domain is negligible. Therefore, the one-way coupling method that transmits information from the fluid domain to the solid domain is selected [10]. In this way, it is necessary to obtain the calculation results of the fluid domain first and apply them as one of the boundary conditions of the solid domain.

**Figure 2:**
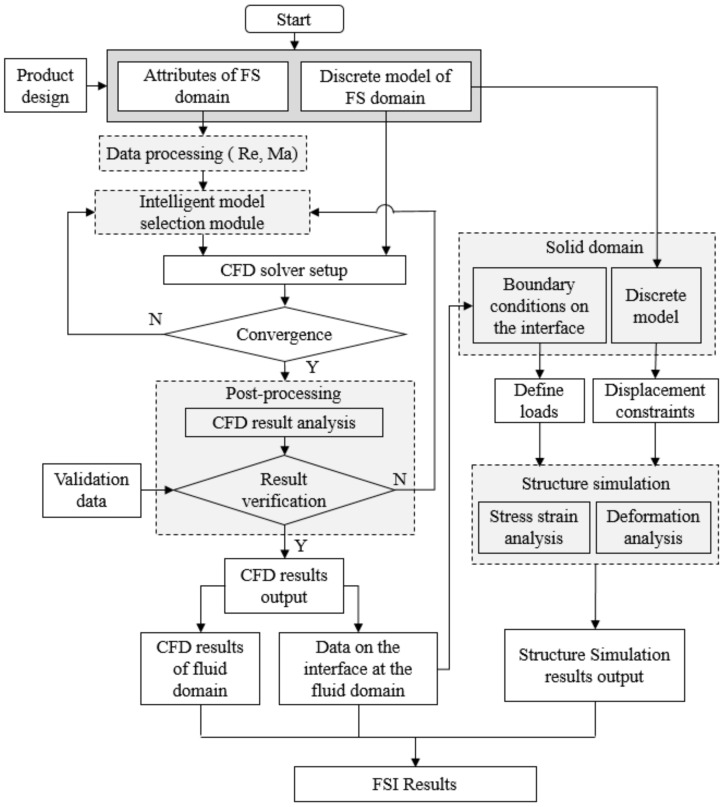
Structure of the intelligent FSI simulation system.

Based on the previous research on the intelligent CFD simulation system [14,39,40], the intelligent FSI simulation system is proposed, as illustrated in Figure 2. This system is composed of CFD simulation and structure simulation, with the interface between FS domains as the medium of the data transfer.

**Figure 3:**
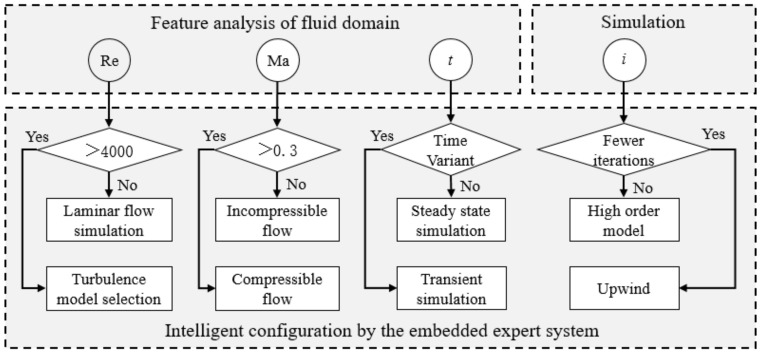
Intelligent model selection module.

Considering the characteristics of AM, more parameters are needed to constrain the flow channel model, which will generate heterogeneous designs to be processed by FSI. The intelligent FSI simulation system is especially suitable for this situation in which there is a large number of FSI simulations of parametric design models. In the proposed system, the attributes of an FS domain can be extracted automatically based on the geometric and physical information conveyed by the flow channel model. Subsequently, the discrete model and the boundary conditions of the FS domain can be configured, respectively. In FSI, CFD is needed to analyze the flow field. However, CFD models require special expertise and rich experience to deal with the nonlinearity, which is at a cost of human resources and great time input. To solve this key issue, an expert system is embedded into the intelligent model selection module of the proposed system to determine the optimal settings and allocate the appropriate fluid physics models according to the design characteristics of different flow channels, as shown in Figure 3. According to the extracted attributes of the input flow channel model, the Reynolds number and the Mach number can be calculated by the following formulas:

(7) Re=ρv¯Dμ

(8) $$\mathrm{Ma}=\frac{\bar{v}}{\sqrt{kRT}}$$

where *ρ* is the density of the fluid, $\bar{v}$ is the average velocity of the fluid, *D* is the inner diameter of the flow channel, *μ* is the dynamic viscosity of the fluid, *k* is the specific heat ratio of the fluid, *R* is the gas constant, and *T* is the temperature of the fluid. Different Reynolds numbers and Mach numbers correspond to different flow regimes that affect the CFD configurations.

Specifically, when the Reynolds number of the flow in channels is greater than 4000, a turbulence model should be selected. When the Mach number is greater than 0.3, selecting the total energy compressible flow model is appropriate. In the meantime, it is necessary that proper boundary conditions and the reference pressure are configured to activate the compressible flow simulation. At the beginning of the simulation, lower order discretization schemes, such as upwind differencing scheme [41] and the *k*–*ε* turbulence model for turbulent flow, should be preferentially selected if the iteration index is small or the simulation has convergence issues. With the increase of the iteration index, higher order advection schemes can be employed to improve the accuracy of simulation. For details, please refer to authors’ previous work published in [39].

After the result validation, the CFD results of the fluid domain and the data associated with the interface are exported. For structural simulation, the boundary condition of the inner wall surface can be obtained by the pressure data exported, while the meshing method of the body and boundary conditions of the fixed end remain unchanged. Then the FSI results are obtained after conducting the structure simulation. Based on this intelligent system, human intervention can be eliminated, and the simulation accuracy can be synchronously guaranteed.

## 4. Intelligent Design Optimization System

### 4.1. System Architecture

**Figure 4:**
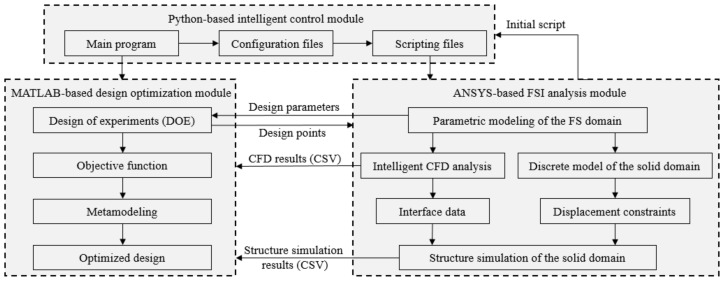
Architecture of the intelligent design optimization system.

Based on the intelligent FSI simulation system proposed in Section 3, the intelligent design optimization system is established, for which the system architecture is shown in Figure 4. The intelligent control module is the basis and guide tool of the whole intelligent design optimization system. The control of the ANSYS-based FSI analysis module and the MATLAB-based design optimization module is realized through the statements which can call each module and the automatic modification of script files. The main program is written in Python to control the simulation and optimization process in an orderly way. In addition, the data transmission and reading between modules also depends on the intelligent control of the main program.

By applying SolidWorks, a parametric CAD model of the fluid domain is established with multiple design variables considering the process constraints of AM. Subsequently, the CAD model of the solid domain with the feature of the wall thickness is built by Boolean operation [42] and the spline interpolation method [43]. All design variables in modeling constitute design points. According to the quantity and the range of design variables, the levels of each design variable and the design points are defined by the design of experiment (DOE) in the design optimization module. The values of levels affect the number of subsequent simulations and the accuracy of mathematical models. The Python-based intelligent control module can create the scripting file corresponding to each design point based on the initial script exported by ANSYS. The main program can call ANSYS to run each scripting file and record all the simulation results into a comma-separated values (CSV) file, which plays the role of the interface between ANSYS and MATLAB. The main program then calls MATLAB to process the CSV file. The FSI-based design optimization is divided into two parts. Firstly, the shape of the fluid domain is optimized based on CFD results recorded in a CSV file. Following that, the optimal value of each design parameter of the solid domain under the constraint is derived based on another CSV file that conveys structure simulation results. The optimized design can be finally acquired if the updated parametric model can be verified by the test simulation.

### 4.2. Principle of the Intelligent System

#### 4.2.1. The Parametric Modeling for AM

**Figure 5:**
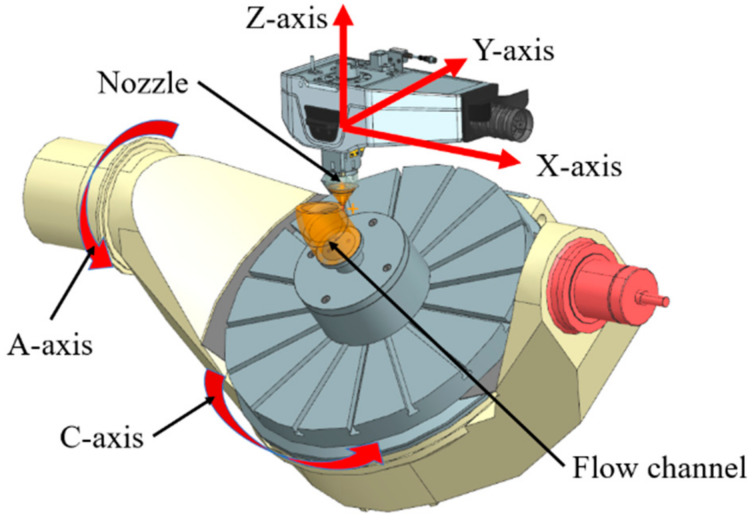
Configuration of the flow channel and major components in a five-axis DED machine tool.

In order to utilize the characteristics of AM and guarantee the manufacturability, the idea of DfAM is introduced into the parametric modeling of flow channels. In this paper, five-axis DED was selected as the AM technology to fabricate flow channels, which is demonstrated in Figure 5. Accordingly, the specific modeling guidelines of flow channels are as follows:

1. The process hole should be replaced by a section of integrated bent channel, which will not only improve the energy efficiency, but also take advantage of AM in producing complex surfaces;
2. The bent section should be designed with variable wall thickness to achieve the goal of weight reduction and satisfy the strength requirement simultaneously;
3. Five-axis DED can obtain flow channels without supporting structures. The premise is that the maximum flow channel bent angle should not exceed 90 degrees to avoid collisions of DED machine tools;
4. Additional geometric constraints should be added to the bent section of the flow channel to smooth the transition between the bent section and the straight section, which guarantees stable machining paths and platform rotation in five-axis DED process;
5. As flow channels belong to thin-walled parts, the wall thickness of its 3D model should be greater than the minimum thickness that can be formed by DED.

#### 4.2.2. FSI Analysis Module

Considering the requirement of intelligent FSI analysis for the solver capability, the method of data exchange, and system integration, ANSYS Workbench was selected as the platform for FSI analysis in this paper. The CFX module and the Static Structural module were applied to conduct the CFD analysis and solid structure analysis, respectively. Mastered by the intelligent control module, the specific steps of intelligent FSI analysis are as follows:

1. The model parameters are input into the main program, and the Reynolds number and Mach number corresponding to different models are calculated based on the aforementioned Equations (7) and (8), respectively;
2. The initial scripting file generated by ANSYS Workbench is used as the source template, and then the source template is updated as new scripting files, which have the same quantity extracted from design models. The configurations of simulation in each new scripting file are also updated by the script statement modification function in the expert system based on the values calculated in the previous step;
3. The new scripting files can run in the specified project one after another calling ANSYS Workbench, which ensures that each design model can be processed automatically;
4. After the FSI simulation is completed, the results corresponding to each design model can be obtained by reading the CSV output file generated by ANSYS.

**Figure 6:**
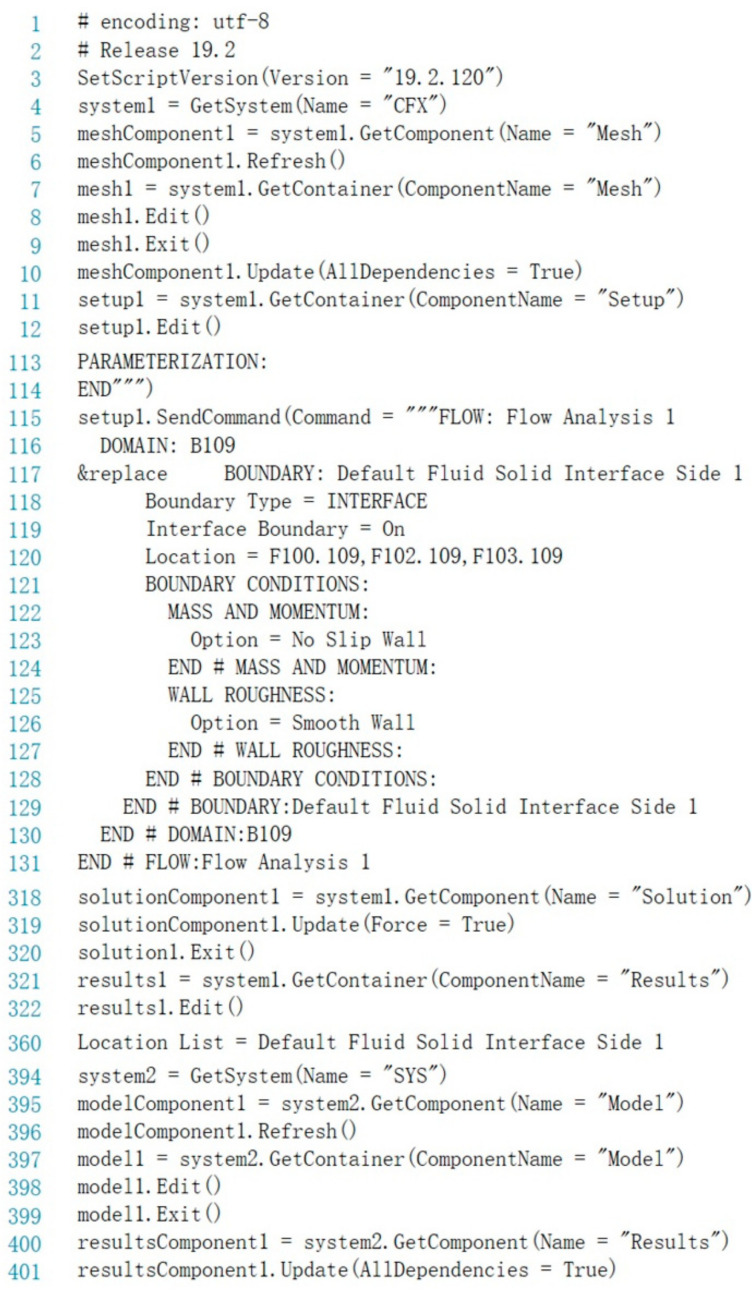
Partial code of the scripting file in the intelligent control module.

It should be noted that the Static Structural module is not compatible with some scripting functions, and thus the data update of the structural simulation cannot be recorded in the generated scripting files automatically. Therefore, a new scripting framework is created to integrate the reading function and the module call function, as shown in Figure 6. Thus, the intelligent simulation and data acquisition of the FSI analysis module can be completely achieved.

#### 4.2.3. Design Optimization Module

Based on metamodeling [44], an approximate relationship between inputs and outputs can be established as a mathematical model that can be used to obtain parameter correlations and predict the value of a certain input. For the design optimization module of the proposed intelligent design optimization system, inputs are the design variables of the parametric model *x* = (*x*_1_, …, *x*_k_), which can be considered as design points [45], while outputs are the simulation results *y* as responses [46]. The collection of all design points and responses serves as a design space. The relationship between *x* and *y* can be described as follows:

(9) $$y=\hat{f}(x,\beta )+\varepsilon$$

where $\hat{f}$(•) is the approximate model, *β* is the coefficient vector, and *ε* is the approximate error. The RSM is applied based on design points *x* and responses *y*. The second-order polynomials are used to derive the approximate objective function as follows:

(10) $$\hat{f}(x_{s},\beta )=\beta_{0}+\sum_{i=1}^{k}\beta_{i}x_{si}+\sum_{i=1}^{k}\beta_{ii}x_{si}^{2}+\sum_{i<j}\sum_{i=1}^{k}\beta_{ij}x_{si}x_{sj}$$

where *k* is the number of design variables in a design point, and *s* is the index of a design point [47]. The design matrix *X* is defined as follows:

(11) $$X=\left[\begin{array}{ccccccc} 1,x_{11}, & \cdots , & x_{1k},x_{11}^{2}, & \cdots , & x_{1k}^{2},x_{11}x_{12}, & \cdots , & x_{1(k-1)}x_{1k}\\ 1,x_{21}, & \cdots , & x_{2k},x_{21}^{2}, & \cdots , & x_{2k}^{2},x_{21}x_{22}, & \cdots , & x_{2(k-1)}x_{2k}\\ \vdots \;\;\;\;\;\vdots \;\;\; & \ddots & \vdots \;\;\;\;\;\;\;\vdots \;\;\; & \ddots & \vdots \;\;\;\;\;\;\;\vdots \;\;\; & \ddots & \vdots \\ 1,x_{n1}, & \cdots , & x_{nk},x_{n1}^{2}, & \cdots , & x_{nk}^{2},x_{n1}x_{n2}, & \cdots , & x_{n(k-1)}x_{nk} \end{array}\right]$$

The coefficients of Equation (9) can be calculated by

(12) $$\beta ={\left(X^{T}X\right)}^{-1}X^{T}y$$

Based on all design variables and simulation results, the objective functions and the constraint equations are established according to Equations (10)–(12), which can predict the response at a given point within the specified range of the design variables. The extreme value can also be obtained by gradient-based optimization in MATLAB. Then it is necessary to run a test FSI simulation of the model corresponding to the extreme value to check whether the error of the extreme value is acceptable compared to the test result. If not, this design point and the corresponding simulation results are added to the design space, and a new round of design optimization will start. The extreme value will not be output as the optimal solution until it is reasonable.

## 5. Case Study

### 5.1. Problem Description

Hydraulic manifolds are typical components with flow channels. The traditional machining method is drilling holes in rectangular metal blocks according to hydraulic schematic diagrams. With the forming principle of layer upon layer superposition, AM can fabricate shapes that traditional processing methods cannot achieve [48], so it is the most suitable processing method for manufacturing the optimized hydraulic manifold.

**Figure 7:**
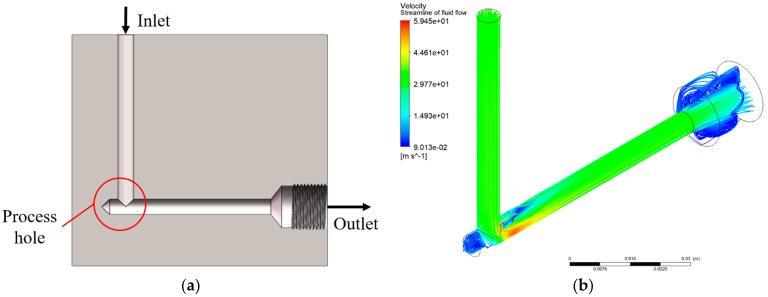
A hydraulic manifold with one flow channel: (**a**) cross-section view of the CAD model, (**b**) streamlines visualized by CFD-Post (Re = 5156 and Ma = 0.019 at the bent 45° cross section).

The purpose of this case study was to optimize the pressure drop and weight of the flow channel without compromising the structural integrity of the manifold by taking advantage of five-axis DED, which is an AM technique that can avoid support structures. As shown in Figure 7a, the hydraulic manifold applied in this study was a simplified geometry composed of one flow channel with a process hole. Verified by simulation, Figure 7b demonstrates that there was a recirculation zone adjacent to the process hole that led to excessive energy loss [49] in this flow channel. Therefore, the optimization objective was to establish the design scheme of the variable cross-section and variable-thickness flow channel with the minimum pressure drop and minimum weight under the strength constraint. The design optimization of the fluid domain and the solid domain was carried out successively.

### 5.2. Design Optimization of the Fluid Domain

#### 5.2.1. Design and Analysis

**Figure 8:**
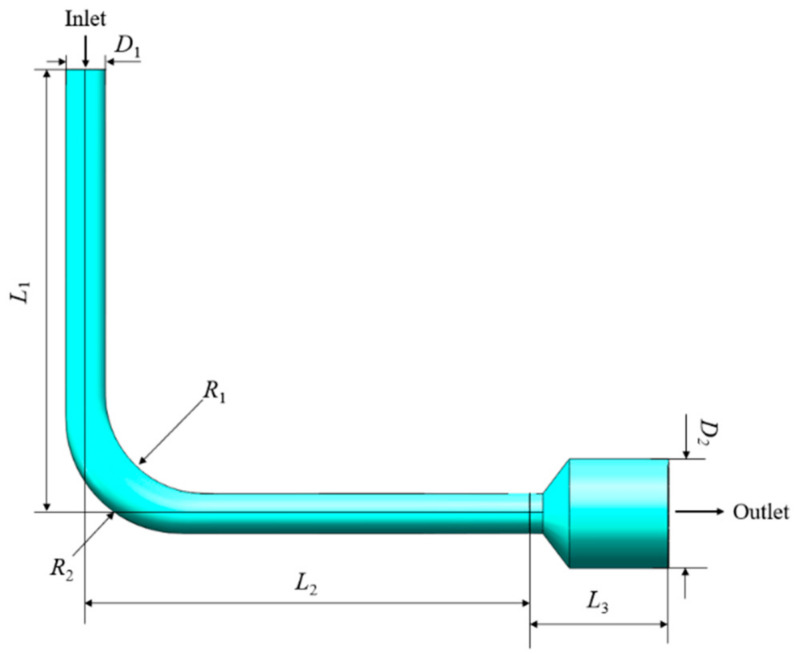
Fluid domain of the flow channel with design variables.

To meet the constraints on the solid design induced by AM, the parametric model of the fluid domain should be a variable cross-section bent flow channel with the same basic dimension parameters as the original design, which are listed in Table 1. As a result, the internal flow domain of the simplified hydraulic manifold shown in Figure 7a was extracted as shown in Figure 8. In this model, *R*_1_ and *R*_2_ were defined as the radius of the inner arc and outer arc of the flow channel, respectively, which were the design variables to be optimized. The goal was to reduce the volume of recirculation zone and obtain the optimal shape of the fluid domain that had the minimum pressure drop.

In this case, the working fluid in the flow channel was L-HL46 hydraulic oil, which has the properties listed in Table 2. The flow velocity at the inlet was set to 35 m/s, and the pressure at the outlet was 20 MPa. Based on DOE, design variables were divided into 5 levels as shown in Table 3. The lowest and highest levels correspond to the lower and upper bounds of design variables, respectively.

#### 5.2.2. Optimization Based on RSM

The numerical experiments were designed by the Central Composite Design (CCD) method, which generated 10 design points. Processed by the intelligent FSI simulation system, the pressure drop values corresponding to each design point were recorded in a CSV file, which is tabulated in Table 4.

Data of all design points in the CSV file were read by the MATLAB-based design optimization module. Based on the data in Table 4, the objective function was derived by RSM as follows:

(13) $$\Delta p=371,290+4010R_{1}-22,080R_{2}+1370R_{1}{}^{2}+1820R_{2}{}^{2}-2760R_{1}R_{2}$$

**Figure 9:**
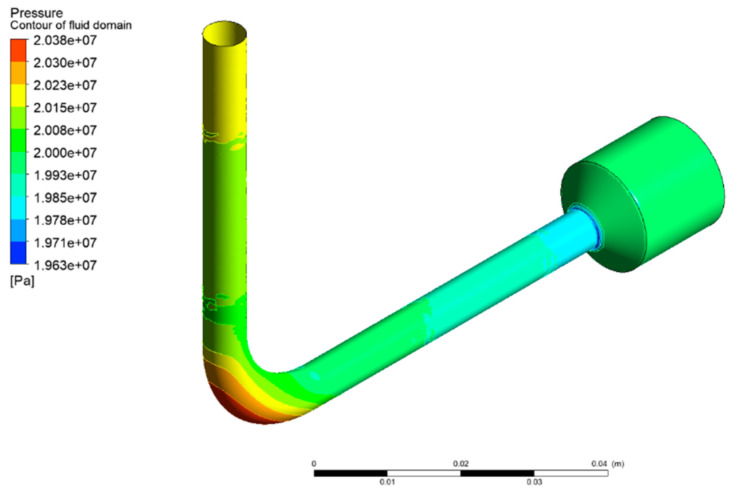
Pressure distribution on the interface of the optimized fluid domain (Re = 5908 and Ma = 0.022 at the bent 45° cross section).

As a result, the minimum pressure drop was 186,600 Pa at the design point (*R*_1_, *R*_2_) = (15, 17.465). This optimal solution was substituted into the parametric model for simulation verification. The simulation result demonstrated that the relative error of the pressure drop was 0.69%, and the optimized pressure drop was 73.43% lower than that of the original design shown in Figure 7a. Figure 9 depicts the pressure distribution on the interface of the optimized fluid domain. It is notable that the maximum pressure appeared at the outer arc of the flow channel.

### 5.3. Optimization of the Solid Domain

#### 5.3.1. Design and Analysis

**Figure 10:**
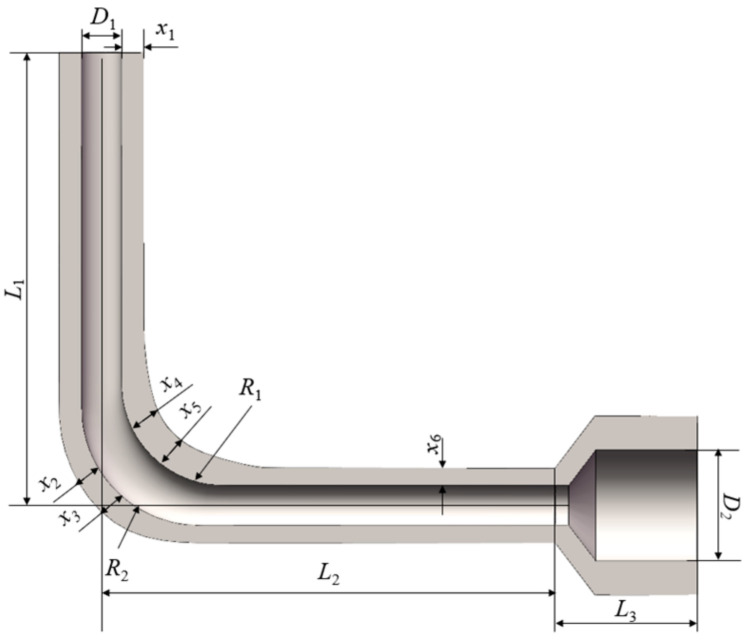
Solid domain of the hydraulic manifold with design variables.

The stress of the arc section was larger compared to the other sections, so the design of local thickness was necessary. According to the optimized fluid domain, a parametric model of the solid domain was established based on the spline interpolation method. As shown in Figure 10, 6 design variables were applied to control the wall thicknesses of four sections of the flow channel: the wall thickness of the vertical section *x*_1_, the wall thicknesses of the outer arc section *x*_2_ and *x*_3_, the wall thicknesses of the inner arc section *x*_4_, and *x*_5_, and the wall thickness of the horizontal section *x*_6_. The goal was to acquire a solid model that had the minimum weight under the strength constraint by controlling the wall thicknesses of each section.

The levels and corresponding values of design variables for the optimization of the solid domain are listed in Table 5. The 316 L stainless steel, common metal AM material, was used as the material, which had a density of 7980 kg/m^3^ and yield strength of 250 MPa, in the structural simulation [50]. It should be noted that the shear stress induced by the fluid on the wall was four orders of magnitude lower than the normal stress caused by the pressure. Therefore, the shear stress was neglected in the application of the interface boundary condition. Then, the maximum strain and stress could be obtained by the structural simulation. Since volume is proportional to weight and could be acquired by SolidWorks directly, it was applied as one of the responses in metamodeling.

#### 5.3.2. Optimization Based on RSM

**Figure 11:**
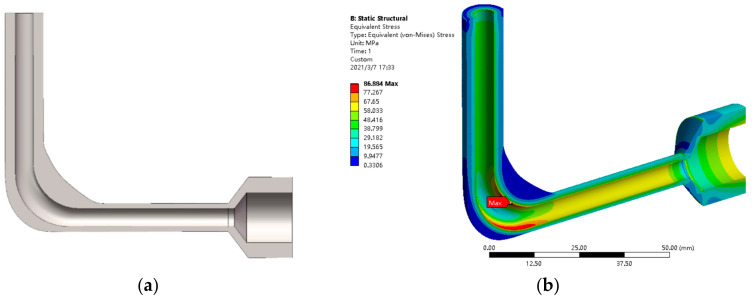
Design optimization of the solid domain: (**a**) cross-sectional view of the CAD model, (**b**) stress distribution.

According to CCD, there were 90 design points generated, which had to be processed by the intelligent FSI simulation system. The values of design variables and the FSI simulation results of each design point are shown in Table A1 of the Appendix A. Based on the data of 90 design points, the objective function was derived by RSM as follows:

(14) $$\begin{array}{ll} V= & 5.4829+1.1008x_{1}+0.0471x_{2}+0.0247x_{3}+0.0387x_{4}+0.067x_{5}+1.5174x_{6}+0.1687x_{1}{}^{2}-\\ & 0.001x_{2}{}^{2}+0.0012x_{3}{}^{2}-0.0021x_{4}{}^{2}-0.0016x_{5}{}^{2}+0.1507x_{6}{}^{2}+0.01x_{1}x_{2}+0.0049x_{1}x_{3}+\\ & 0.016x_{1}x_{4}+0.0032x_{1}x_{5}+0.0106x_{1}x_{6}+0.002x_{2}x_{3}-0.0001x_{2}x_{4}-0.0015x_{2}x_{5}+\\ & 0.0058x_{2}x_{6}+0.0013x_{3}x_{4}+0.0024x_{3}x_{5}+0.0072x_{3}x_{6}-0.0006x_{4}x_{5}+0.0046x_{4}x_{6}+0.0062x_{5}x_{6} \end{array}$$

and the constraint equation was also derived as follows:

(15) $$\begin{array}{ll} \sigma_{\mathrm{Max}}= & 223.0129-30.6255x_{1}-1.6095x_{2}-2.5868x_{3}-6.1686x_{4}-5.7091x_{5}-18.937x_{6}+\\ & 1.8943x_{1}{}^{2}-0.2387x_{2}{}^{2}+0.2731x_{3}{}^{2}+0.1103x_{4}{}^{2}+0.0863x_{5}{}^{2}+0.4902x_{6}{}^{2}+0.0358x_{1}x_{2}+\\ & 0.1807x_{1}x_{3}+0.7707x_{1}x_{4}+0.2898x_{1}x_{5}+1.6479x_{1}x_{6}+0.1054x_{2}x_{3}-0.0982x_{2}x_{4}-\\ & 0.0585x_{2}x_{5}+0.0211x_{2}x_{6}-0.0161x_{3}x_{4}-0.1031x_{3}x_{5}+0.0856x_{3}x_{6}+0.2145x_{4}x_{5}+\\ & 0.2843x_{4}x_{6}+\;0.7467x_{5}x_{6}\leq \sigma_{s}/n \end{array}$$

where *σ*_s_ is the yield strength, and *n* is the factor of safety, which was equal to 3 in this case. Applying the gradient method, the optimal combination of variables *x*_1_ to *x*_6_ under the strength constraint was 3.2488, 4.1469, 3.704, 7.37, 7.37, and 1.58, with the minimum volume being 15,460 mm^3^. This optimal solution was substituted into the parametric model, as shown in Figure 11a. By simulation, it was verified that the relative error of the maximum stress of the optimized design was 3.43%, and the relative error of volume was less than 0.001%, which all met the accuracy requirement.

**Figure 12:**
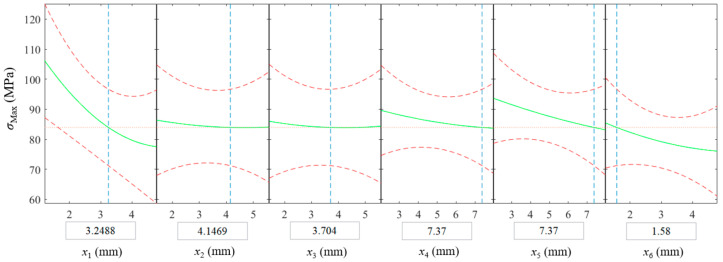
Influence of the design variables on the maximum stress.

The structural simulation result of the optimized design is shown in Figure 11b. Compared with the model in Figure 7a, the volume of the flow channel was reduced by 90.53%. Moreover, the volume was 20.66% lower than that of the flow channel optimized with uniform wall thickness. Figure 12 depicts the influence of design variables on the maximum stress. Evidently, the maximum stress was most sensitive to the wall thickness of the vertical pipe section *x*_1_. According to the strain contour gained from the structural simulation, the flow channel had a tendency to bend inward under the flow pressure, resulting in a greater stress in the inner arc section than the outer arc section. The optimization program enhanced the inner arc section to provide sufficient support, resulting in the fact that the wall thicknesses of the outer arc section *x*_2_ and *x*_3_ had almost no effect on the maximum stress value.

### 5.4. Manufacturing of the Optimized Flow Channel

**Figure 13:**
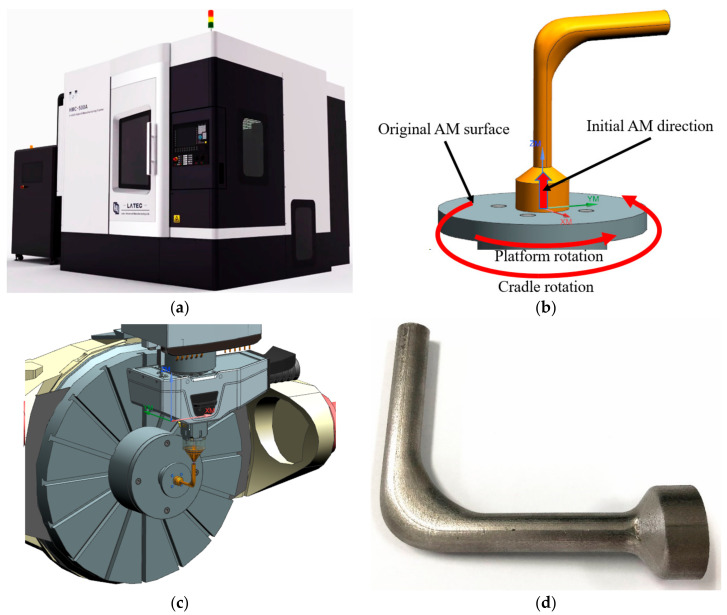
The optimized flow channel manufactured by five-axis DED: (**a**) LATEC HMC-500A, (**b**) AM process planning, (**c**) manufacturing process simulation, (**d**) manufactured part.

To verify the applicability of the intelligent design optimization system to AM, the optimized flow channel was manufactured by LATEC HMC-500A, which is a cradle five-axis DED machine tool shown in Figure 13a. As shown in Figure 13b, the end face of the outlet was chosen as the original AM surface, and the direction perpendicular to this surface was determined as the initial AM direction. Facilitated by the five-axis capability, it allows the flow channel part to be manufactured along its axis to avoid support structures. The manufacturing of the bent channel section was realized by the rotation of the platform and the cradle according to the curved axis of this section. The 316 L stainless steel powder was applied as the material for AM. The minimum wall thickness of the flow channel was 1.58 mm, greater than the DED minimum wall thickness requirement of 1 mm. The process parameters were set in the dialog box of the machine tool control system according to the material properties and dimensions of the model. Then the execution of the part fabrication was checked by the manufacturing process simulation in the environment of the machine tool. Figure 13c shows the processing position where the cradle rotates 90 degrees.

After AM, milling and abrasive blasting were carried out on the outer surface of the flow channel structure, and the final part is shown in Figure 13d. Therefore, the proposed intelligent design optimization system derives an improved flow channel with satisfactory manufacturability under the DfAM framework.

## 6. Conclusions

In this paper, an integrated design optimization system is proposed, which is composed of a Python-based intelligent control module, an ANSYS-based FSI analysis module, and a MATLAB-based design optimization module. The operation of these modules is realized by the manipulated script file and data transmission. DfAM of flow channels was implemented by considering the characteristics and process constraints of AM in the modeling process. An expert system is embedded in the intelligent control module, so that the FSI analysis module can be called based on the scripting files to realize the intelligent simulation of the models corresponding to different design points created by DOE. The simulation results are obtained after the solving process. Based on metamodeling, the approximate relationship between design variables and properties of the flow channel is established to derive the optimal solution by optimization algorithms.

The effectiveness of this system is verified by the design optimization of a simplified hydraulic manifold with one flow channel. Based on the proposed system, the design variables and simulation results corresponding to each design point are obtained and recorded in a CSV file. Based on RSM and gradient-based optimization, the optimized design with minimum pressure drops and minimum volume is derived under the strength constraints. The successful manufacturing of the optimized flow channel part demonstrates that the proposed approach has achieved DfAM by considering the processing requirements of AM in design optimization.

At present, the intelligent design optimization system has only demonstrated its capability in the optimization and the AM of a simplified hydraulic manifold. In the future, the optimized flow channel will be manufactured by additive–subtractive hybrid manufacturing, which can guarantee the machining quality of the inner surface, and thus the optimization effect can be tested by experiment. In addition, this system will be further developed to optimize hydraulic manifolds with multi-channels to verify its applicability in practical scenarios.

## Figures and Tables

**Table 1:** The values of dimension parameters.

Parameter	Value (mm)
*D*_1_	6
*D*_2_	16.5
*L*_1_	67
*L*_2_	67
*L*_3_	21

**Table 2:** The values of parameters associated with the working fluid.

Parameter	Value	Unit
*T*	323.15	K
*p*	20	MPa
*ρ*	870.15	kg/m^3^
*C*	4812	Cal/(g·K)
*μ*	0.02501	kg/(m s)

**Table 3:** Levels of design variables of the fluid domain.

Level	*R*_1_ (mm)	*R*_2_ (mm)
1.828	14.398	20.398
1	11.5	17.5
0	8	14
−1	4.5	10.5
−1.828	1.602	7.602

**Table 4:** Design points with pressure drop.

Design Point	*R*_1_ (mm)	*R*_2_ (mm)	Δ*p* (Pa)
1	4.5	10.5	251,030
2	4.5	17.5	366,420
3	11.5	10.5	234,500
4	11.5	17.5	214,860
5	1.6	14	369,470
6	14.4	14	203,640
7	8	7.6	262,250
8	8	20.4	347,080
9	8	14	230,700
10	8	14	230,700

**Table 5:** Levels of design variables of the solid domain.

Level	*x*_1_ (mm)	*x*_2_ (mm)	*x*_3_ (mm)	*x*_4_ (mm)	*x*_5_ (mm)	*x*_6_ (mm)
2.828	4.7	5.48	5.48	7.83	7.83	4.7
1	3.6	4.2	4.2	6	6	3.6
0	3	3.5	3.5	5	5	3
−1	2.4	2.8	2.8	4	4	2.4
−2.828	1.3	1.52	1.52	2.17	2.17	1.3

## Appendix A

**Table A1:** Design points with volume and maximum stress.

Design Point	*x*_1_ (mm)	*x*_2_ (mm)	*x*_3_ (mm)	*x*_4_ (mm)	*x*_5_ (mm)	*x*_6_ (mm)	Volume (mm^3^)	Maximum Stress (MPa)
1	3.6	4.2	4.2	6	4	2.4	17,897.476	83.875
2	3.6	4.2	4.2	6	4	3.6	20,968.032	77.451
3	3.6	4.2	4.2	6	6	2.4	18,048.811	80.785
4	3.6	4.2	4.2	6	6	3.6	21,152.207	78.199
5	3.6	4.2	4.2	4	6	2.4	17,875.377	82.833
6	3.6	4.2	4.2	4	6	3.6	20,966.183	77.099
7	3.6	4.2	4.2	4	4	2.4	17,710.653	86.241
8	3.6	4.2	4.2	4	4	3.6	20,786.965	79.586
9	3.6	4.2	2.8	6	4	2.4	17,771.738	83.405
10	3.6	4.2	2.8	6	4	3.6	20,848.548	77.13
11	3.6	4.2	2.8	6	6	2.4	17,902.091	80.711
12	3.6	4.2	2.8	6	6	3.6	21,002.514	76.702
13	3.6	4.2	2.8	4	6	2.4	17,742.931	82.403
14	3.6	4.2	2.8	4	6	3.6	20,804.92	76.954
15	3.6	4.2	2.8	4	4	2.4	17,587.042	85.323
16	3.6	4.2	2.8	4	4	3.6	20,635.994	78.576
17	3.6	2.8	4.2	6	4	2.4	17,766.073	83.68
18	3.6	2.8	4.2	6	4	3.6	20,830.547	77.7
19	3.6	2.8	4.2	6	6	2.4	17,907.069	80.76
20	3.6	2.8	4.2	6	6	3.6	21,007.847	77.399
21	3.6	2.8	4.2	4	6	2.4	17,737.808	82.23
22	3.6	2.8	4.2	4	6	3.6	20,819.957	77.307
23	3.6	2.8	4.2	4	4	2.4	17,577.73	85.738
24	3.6	2.8	4.2	4	4	3.6	20,636.711	78.545
25	3.6	2.8	2.8	6	4	2.4	17,629.393	83.133
26	3.6	2.8	2.8	6	4	3.6	20,677.002	76.828
27	3.6	2.8	2.8	6	6	2.4	17,789.481	80.755
28	3.6	2.8	2.8	6	6	3.6	20,852.685	77.18
29	3.6	2.8	2.8	4	6	2.4	17,609.845	82.125
30	3.6	2.8	2.8	4	6	3.6	20,682.307	77.352
31	3.6	2.8	2.8	4	4	2.4	17,460.586	84.905
32	3.6	2.8	2.8	4	4	3.6	20,499.801	78.604
33	3	3.5	3.5	5	5	3	17,798.702	84.732
34	3	3.5	3.5	5	5	3	17,798.702	84.732
35	3	3.5	3.5	5	5	3	17,798.702	84.732
36	3	3.5	3.5	5	5	3	17,798.702	84.732
37	3	3.5	3.5	5	5	3	17,798.702	84.732
38	3	3.5	3.5	5	5	3	17,798.702	84.732
39	2.4	4.2	4.2	6	4	2.4	15,119.431	93.836
40	2.4	4.2	4.2	6	4	3.6	18,189.79	85.819
41	2.4	4.2	4.2	6	6	2.4	15,273.54	90.887
42	2.4	4.2	4.2	6	6	3.6	18,349.325	83.995
43	2.4	4.2	4.2	4	6	2.4	15,160.95	93.869
44	2.4	4.2	4.2	4	6	3.6	18,194.569	87.043
45	2.4	4.2	4.2	4	4	2.4	14,972.612	97.894
46	2.4	4.2	4.2	4	4	3.6	18,036.759	89.345
47	2.4	4.2	2.8	6	4	2.4	15,002.218	93.788
48	2.4	4.2	2.8	6	4	3.6	18,048.514	85.408
49	2.4	4.2	2.8	6	6	2.4	15,145.882	90.865
50	2.4	4.2	2.8	6	6	3.6	18,206.417	84.055
51	2.4	4.2	2.8	4	6	2.4	14,998.914	93.891
52	2.4	4.2	2.8	4	6	3.6	18,060.763	86.783
53	2.4	4.2	2.8	4	4	2.4	14,857.127	97.363
54	2.4	4.2	2.8	4	4	3.6	17,929.129	88.834
55	2.4	2.8	4.2	6	4	2.4	15,005.44	93.953
56	2.4	2.8	4.2	6	4	3.6	18,067.713	85.678
57	2.4	2.8	4.2	6	6	2.4	15,150.937	90.986
58	2.4	2.8	4.2	6	6	3.6	18,230.22	84.009
59	2.4	2.8	4.2	4	6	2.4	15,011.626	93.745
60	2.4	2.8	4.2	4	6	3.6	18,064.973	86.61
61	2.4	2.8	4.2	4	4	2.4	14,863.887	97.405
62	2.4	2.8	4.2	4	4	3.6	17,910.394	88.809
63	2.4	2.8	2.8	6	4	2.4	14,878.343	93.982
64	2.4	2.8	2.8	6	4	3.6	17,918.057	85.383
65	2.4	2.8	2.8	6	6	2.4	15,037.007	91.242
66	2.4	2.8	2.8	6	6	3.6	18,098.979	83.893
67	2.4	2.8	2.8	4	6	2.4	14,890.674	93.835
68	2.4	2.8	2.8	4	6	3.6	17,944.428	86.406
69	2.4	2.8	2.8	4	4	2.4	14,746.24	97.228
70	2.4	2.8	2.8	4	4	3.6	17,778.339	88.442
71	3	3.5	3.5	5	5	3	17,798.702	84.732
72	3	3.5	3.5	5	5	3	17,798.702	84.732
73	1.3	3.5	3.5	5	5	3	14,385.117	105.84
74	4.7	3.5	3.5	5	5	3	22,185.297	76.622
75	3	1.52	3.5	5	5	3	17,614.667	89.408
76	3	5.48	3.5	5	5	3	17,980.02	83.986
77	3	3.5	1.52	5	5	3	17,614.18	89.593
78	3	3.5	5.48	5	5	3	17,990.502	84.071
79	3	3.5	3.5	2.17	5	3	17,562.815	91.662
80	3	3.5	3.5	7.83	5	3	17,998.965	81.625
81	3	3.5	3.5	5	2.17	3	17,559.502	91.296
82	3	3.5	3.5	5	7.83	3	18,010.566	81.606
83	3	3.5	3.5	5	5	1.3	13,899.297	96.837
84	3	3.5	3.5	5	5	4.7	22,567.122	77.509
85	3	3.5	3.5	5	5	3	17,798.702	84.732
86	3	3.5	3.5	5	5	3	17,798.702	84.732
87	3	3.5	3.5	5	5	3	17,798.702	84.732
88	3	3.5	3.5	5	5	3	17,798.702	84.732
89	3	3.5	3.5	5	5	3	17,798.702	84.732
90	3	3.5	3.5	5	5	3	17,798.702	84.732
